# State-Specific Severe Joint Pain and Physical Inactivity Among Adults with Arthritis — United States, 2017

**DOI:** 10.15585/mmwr.mm6817a2

**Published:** 2019-05-03

**Authors:** Dana Guglielmo, Louise B. Murphy, Michael A. Boring, Kristina A. Theis, Charles G. Helmick, Jennifer M. Hootman, Erica L. Odom, Susan A. Carlson, Yong Liu, Hua Lu, Janet B. Croft

**Affiliations:** ^1^Division of Population Health, National Center for Chronic Disease Prevention and Health Promotion, CDC; ^2^Oak Ridge Institute for Science and Education, Oak Ridge, Tennessee; ^3^Division of Nutrition, Physical Activity and Obesity, National Center for Chronic Disease Prevention and Health Promotion, CDC.

An estimated 54.4 million (approximately one in four) U.S. adults have doctor-diagnosed arthritis (arthritis) (*1*). Severe joint pain and physical inactivity are common among adults with arthritis and are linked to adverse mental and physical health effects and limitations (*2*,*3*). CDC analyzed 2017 Behavioral Risk Factor Surveillance System (BRFSS) data to estimate current state-specific prevalence of arthritis and, among adults with arthritis, the prevalences of severe joint pain and physical inactivity. In 2017, the median age-standardized state prevalence of arthritis among adults aged ≥18 years was 22.8% (range = 15.7% [District of Columbia] to 34.6% [West Virginia]) and was generally highest in Appalachia and Lower Mississippi Valley regions.* Among adults with arthritis, age-standardized, state-specific prevalences of both severe joint pain (median = 30.3%; range = 20.8% [Colorado] to 45.2% [Mississippi]) and physical inactivity (median = 33.7%; range = 23.2% [Colorado] to 44.4% [Kentucky]) were highest in southeastern states. Physical inactivity prevalence among those with severe joint pain (47.0%) was higher than that among those with moderate (31.8%) or no/mild joint pain (22.6%). Self-management strategies such as maintaining a healthy weight or being physically active can reduce arthritis pain and prevent or delay arthritis-related disability. Evidence-based physical activity and self-management education programs are available that can improve quality of life among adults with arthritis.

BRFSS is an ongoing state-based, landline and cellular telephone survey of noninstitutionalized adults in the United States aged ≥18 years that is conducted by state and territorial health departments in 50 U.S. states, the District of Columbia (DC), and U.S. territories.^†^ The combined (telephone and cellular) median response rate in 2017 among states was 45.9% (range = 30.6%–64.1%); 435,331 adults reported information about arthritis status and age, and among them, 144,099 reported having arthritis.^§^ Having arthritis was defined as a response of “yes” to the question “Have you ever been told by a doctor or other health care professional that you have arthritis, rheumatoid arthritis, gout, lupus, or fibromyalgia?” No/mild, moderate, and severe joint pain were defined by responses of 0–3, 4–6, and 7–10, respectively, to the question “Please think about the past 30 days, keeping in mind all of your joint pain or aching and whether or not you have taken medication. On a scale of 0 to 10 where 0 is no pain or aching and 10 is pain or aching as bad as it can be, during the past 30 days, how bad was your joint pain on average?” Physical inactivity was defined as a response of “no” to the question “During the past month, other than your regular job, did you participate in any physical activities or exercises such as running, calisthenics, golf, gardening, or walking for exercise?”

All analyses, which accounted for BRFSS’s complex sampling design, were conducted using SAS (version 9.4; SAS Institute) and SUDAAN (version 11.0; RTI International). Sampling weights, using iterative proportional fitting (raking), were applied to make estimates representative of each state.^¶^ Age-standardized,** state-specific prevalences of arthritis among adults aged ≥18 years, and of severe joint pain and physical inactivity among adults with arthritis, were calculated by selected characteristics. Differences across subgroups were tested using t-tests, and orthogonal linear contrasts were conducted for tests of trends to detect linear patterns in ordinal variables (*4*); all differences and trends reported in the text are significant (α = 0.05).

In 2017, age-specific arthritis prevalence was higher with increasing age, ranging from 8.1% among those aged 18–44 years to 50.4% among those aged ≥65 years (Table 1). Age-standardized arthritis prevalence was significantly higher among women (25.4%) than among men (19.1%); non-Hispanic American Indian/Alaska Natives (29.7%) than among other racial/ethnic groups (range = 12.8%–25.5%); and those unable to work/disabled (51.3%), compared with retired (34.3%), unemployed (26.0%), or employed/self-employed (17.7%). Arthritis prevalence was higher with increasing body mass index, ranging from 17.9% among those with healthy weight or underweight to 30.4% among those with obesity. Arthritis prevalence was lower among Hispanics and non-Hispanic Asians than among other racial/ethnic groups, was inversely related to education and federal poverty level, and was higher among those living in more rural areas compared with urban dwellers.

**TABLE 1:** Age-specific and age-standardized* prevalence of arthritis^†^ among U.S. adults aged ≥18 years, and among those with arthritis, prevalences of severe joint pain,^§^ and physical inactivity,^¶^ by selected characteristics — Behavioral Risk Factor Surveillance System, United States, 2017

Characteristic	Sample size (adults aged ≥18 yrs)	Unweighted no. with arthritis**	Arthritis, % (95% CI)	Severe joint pain,^††^ % (95% CI)	Physical inactivity,^††^ % (95% CI)
**Age group (yrs)**					
18–44	122,340	11,615	8.1 (7.8–8.3)	33.0 (31.3–34.7)	31.0 (29.4–32.7)
45–64	159,379	54,383	31.8 (31.3–32.3)	35.6 (34.7–36.5)	35.9 (35.0–36.8)
≥65	153,612	78,101	50.4 (49.8–51.0)	25.1 (24.3–25.9)	37.0 (36.1–37.8)
**Sex**					
Men	192,681	52,827	19.1 (18.8–19.4)	27.3 (25.9–28.7)	30.4 (29.1–31.7)
Women	242,460	91,221	25.4 (25.0–25.7)	36.0 (34.7–37.3)	35.6 (34.3–36.9)
**Race/Hispanic ethnicity^§§^**					
White	331,585	116,255	24.1 (23.8–24.3)	27.4 (26.4–28.4)	31.8 (30.8–32.8)
Black	34,952	11,594	24.1 (23.3–24.9)	50.9 (48.0–53.9)	40.4 (37.3–43.5)
Hispanic	32,064	5,800	16.9 (16.2–17.7)	42.0 (38.7–45.4)	36.0 (32.8–39.3)
Asian	9,165	1,161	12.8 (11.2–14.5)	27.7 (16.9–41.8)^¶¶^	36.1 (25.0–48.9)
American Indian/Alaska Native	8,206	2,805	29.7 (27.2–32.4)	42.0 (35.3–49.0)	33.2 (27.7–39.1)
Other/Multiple race	11,952	3,930	25.5 (24.1–27.0)	37.4 (33.4–41.7)	33.3 (29.1–37.7)
**Highest level of education**					
Less than high school graduate	31,177	12,595	25.7 (24.9–26.6)	54.1 (51.0–57.2)	46.4 (43.1–49.6)
High school graduate or equivalent	118,840	43,212	23.4 (23.0–23.8)	35.5 (33.9–37.1)	38.7 (37.0–40.3)
Technical school/Some college	120,950	42,634	24.4 (23.9–24.8)	30.2 (28.5–31.9)	31.6 (30.1–33.2)
College degree or higher	163,230	45,317	17.5 (17.1–17.8)	15.1 (14.0–16.3)	20.0 (18.7–21.4)
**Employment status**					
Employed/Self-employed	217,384	44,544	17.7 (17.4–18.1)	20.6 (19.5–21.8)	29.2 (28.0–30.4)
Unemployed	18,884	5,864	26.0 (24.9–27.2)	39.9 (36.6–43.3)	33.4 (30.4–36.7)
Retired	129,618	64,620	34.3 (28.4–40.7)	45.8 (35.0–57.1)	31.1 (24.2–39.1)
Unable to work/Disabled	31,689	20,443	51.3 (49.8–52.7)	66.9 (64.9–68.9)	51.2 (48.8–53.5)
Other	34,662	7,965	21.1 (20.2–22.0)	30.6 (27.3–34.2)	29.4 (26.0–32.9)
Federal poverty level***					
≤125% FPL	59,064	23,120	28.6 (28.0–29.3)	51.6 (49.6–53.6)	42.6 (40.6–44.7)
>125% to ≤200% FPL	55,134	22,702	24.7 (24.0–25.5)	33.0 (30.5–35.5)	36.7 (33.9–39.5)
>200% to ≤400% FPL	89,104	32,172	22.4 (21.9–23.0)	24.9 (22.6–27.3)	31.1 (28.8–33.4)
>400% FPL	117,078	30,457	18.4 (17.9–18.8)	13.9 (12.0–16.1)	20.7 (18.8–22.6)
**Sexual orientation^†††^**					
Straight	185,994	63,300	22.1 (21.8–22.5)	31.7 (30.1–33.3)	33.4 (32.0–34.9)
Lesbian/Gay/Bisexual/Queer/ Questioning	9,346	2,646	22.5 (21.1–24.0)	40.7 (36.3–45.4)	33.2 (29.2–37.5)
**Urban-rural status^§§§^**					
Large metro center	68,712	18,857	19.5 (19.0–20.0)	34.2 (31.5–37.0)	30.7 (28.2–33.3)
Large fringe metro	83,056	26,913	22.2 (21.7–22.6)	28.6 (26.7–30.6)	31.6 (29.7–33.6)
Medium metro	90,803	29,572	23.1 (22.7–23.5)	33.0 (31.3–34.7)	34.0 (32.3–35.8)
Small metro	60,652	20,685	24.0 (23.5–24.6)	32.7 (30.8–34.7)	35.0 (33.0–37.1)
Micropolitan	65,752	23,315	26.3 (25.6–26.9)	33.3 (30.9–35.7)	37.0 (34.7–39.4)
Rural (noncore)	66,356	24,757	27.7 (26.9–28.5)	35.7 (33.2–38.3)	38.7 (36.2–41.2)
**Body mass index (kg/m^2^)**					
Underweight/Healthy weight (<25)	131,890	34,818	17.9 (17.5–18.2)	29.1 (27.2–31.0)	28.6 (27.0–30.3)
Overweight (25 to <30)	145,099	46,441	20.4 (20.0–20.8)	28.6 (26.7–30.5)	27.8 (26.2–29.5)
Obese (≥30)	125,421	53,342	30.4 (29.9–30.9)	37.2 (35.7–38.7)	39.3 (37.7–40.8)

**Abbreviations:** CI = confidence interval; FPL = federal poverty level.

* Except for age groups, estimates were age-standardized to the 2000 projected U.S. population aged ≥18 years using three groups (18–44, 45–64, and ≥65 years): https://www.cdc.gov/nchs/data/statnt/statnt20.pdf.

^†^ Respondents were classified as having arthritis if they responded “yes” to the question “Have you ever been told by a doctor or other health care professional that you have arthritis, rheumatoid arthritis, gout, lupus, or fibromyalgia?” Overall, 144,099 respondents reported arthritis.

^§^ Severe joint pain was defined as a response of 7–10 to “Please think about the past 30 days, keeping in mind all of your joint pain or aching and whether or not you have taken medication. On a scale of 0 to 10 where 0 is no pain or aching and 10 is pain or aching as bad as it can be, during the past 30 days, how bad was your joint pain on average?” Overall, 141,744 (98.4%) respondents with arthritis had severe joint pain data available.

^¶^ Physical inactivity was defined as reporting “no” to the question “During the past month, other than your regular job, did you participate in any physical activities or exercises such as running, calisthenics, golf, gardening, or walking for exercise?” Overall, 135,160 (93.8%) respondents with arthritis had physical inactivity data available.

** Categories might not sum to sample total because of missing responses for some variables.

^††^ Among adults aged ≥18 years with arthritis.

^§§^ Persons who identified as Hispanic might be of any race. Persons who identified with a racial group were all non-Hispanic.

^¶¶^ Estimate is potentially unreliable because the relative standard error was between 20% and 30%.

*** Federal poverty level is the ratio of total family income to federal poverty level per family size. Overall, 35,648 respondents had missing data.

^†††^ Sexual orientation was not asked in every state. The 27 states that asked sexual orientation were California, Connecticut, Delaware, Florida, Georgia, Hawaii, Illinois, Indiana, Iowa, Louisiana, Massachusetts, Minnesota, Mississippi, Montana, Nevada, New York, North Carolina, Ohio, Oklahoma, Pennsylvania, Rhode Island, South Carolina, Texas, Vermont, Virginia, Washington, and Wisconsin. A total of 1,049 respondents refused to answer.

^§§§^ Urban-rural status was categorized using the National Center for Health Statistics 2013 Urban-Rural Classification Scheme for Counties: https://www.cdc.gov/nchs/data/series/sr_02/sr02_166.pdf.

Among adults with arthritis, no/mild, moderate, and severe joint pain was reported by 36.2% (95% confidence interval [CI] = 35.7%–36.8%), 33.0% (CI = 32.4%–33.5%), and 30.8% (CI = 30.3%–31.4%) of respondents, respectively (unadjusted prevalences). Age-specific percentages for severe joint pain declined with increasing age, ranging from 33.0% among those aged 18–44 years to 25.1% among those aged ≥65 years. Age-standardized severe joint pain prevalence was ≥40% among the following groups: those unable to work/disabled (66.9%); those with less than a high school diploma (54.1%); those living at ≤125% federal poverty level (51.6%); non-Hispanic blacks (50.9%); retired persons (45.8%); Hispanics (42.0%); non-Hispanic American Indians/Alaska Natives (42.0%); and lesbian/gay/bisexual/queer/questioning (40.7%; reported by 27 states). Severe joint pain prevalence was similar across urban/rural geographic areas, ranging from 32.7%–35.7% in all areas, except for a lower prevalence (28.6%) in large fringe metro areas (Table 1).

Among adults with arthritis, age-specific physical inactivity prevalence was higher with increasing age (ranging from 31.0% among those aged 18–44 years to 37.0% among those aged ≥65 years). Age-standardized physical inactivity prevalence was ≥40% among the following groups: those unable to work/disabled (51.2%); those with less than a high school diploma (46.4%); those living at ≤125% federal poverty level (42.6%); and non-Hispanic blacks (40.4%). Physical inactivity prevalence increased with increasing rurality and with increasing joint pain levels (ranging from 22.6% among those with no/mild joint pain to 47.0% among those with severe joint pain).

Median age-standardized state prevalence of arthritis among adults aged ≥18 years was 22.8% (range = 15.7% [DC] to 34.6% [West Virginia]) (Table 2) and was highest in Appalachia and Lower Mississippi Valley regions. Among 144,099 adults with arthritis, median age-standardized state prevalences of severe joint pain and physical inactivity were 30.3% (range = 20.8% [Colorado] to 45.2% [Mississippi]) and 33.7% (range = 23.2% [Colorado] to 44.4% [Kentucky]), respectively. Age-standardized severe joint pain (Figure) and physical inactivity prevalences were highest in southeastern states.

**TABLE 2:** State-specific crude and age-standardized* prevalence of arthritis^†^ among U.S. adults aged ≥18 years and among those with arthritis, prevalences of severe joint pain,^§^ and physical inactivity^¶^ — Behavioral Risk Factor Surveillance System, United States, 2017

State	Arthritis				Severe joint pain**				Physical inactivity**			
	No.		Prevalence, % (95% CI)		No.		Prevalence, % (95% CI)		No.		Prevalence, % (95% CI)	
	Unweighted	Weighted (x 1,000)	Crude	Age-standardized	Unweighted	Weighted (x 1,000)	Crude	Age-standardized	Unweighted	Weighted (x 1,000)	Crude	Age-standardized
Alabama	2,778	1,241	33.3 (31.9–34.8)	30.4 (29.0–31.8)	1,050	477	38.9 (36.5–41.4)	41.1 (37.0–45.3)	1,158	519	44.6 (42.1–47.2)	42.3 (38.1–46.6)
Alaska	943	124	22.5 (20.4–24.8)	22.8 (20.8–25.0)	193	28	22.6 (18.2–27.7)	23.8 (17.1–32.1)	267	34	29.0 (24.2–34.3)	29.1 (21.8–37.8)
Arizona	4,925	1,285	24.3 (23.5–25.1)	22.0 (21.3–22.8)	1,248	384	30.3 (28.6–32.1)	31.7 (28.6–35.0)	1,472	405	34.5 (32.7–36.3)	29.3 (26.4–32.4)
Arkansas	2,298	700	30.9 (28.8–33.0)	28.4 (26.3–30.5)	734	260	37.6 (33.9–41.5)	42.4 (35.8–49.3)	920	265	40.6 (36.9–44.3)	36.8 (30.8–43.2)
California	2,095	5,873	19.5 (18.4–20.6)	18.3 (17.4–19.3)	547	1,682	28.9 (26.1–31.8)	29.7 (24.6–35.3)	461	1,432	26.6 (23.8–29.5)	26.3 (21.8–31.3)
Colorado	2,796	920	21.4 (20.5–22.3)	20.3 (19.5–21.1)	500	188	20.8 (19.0–22.8)	20.8 (17.8–24.3)	578	200	24.5 (22.4–26.7)	23.2 (19.7–27.2)
Connecticut	3,269	639	23.1 (22.1–24.1)	20.1 (19.3–21.0)	707	161	25.6 (23.5–27.9)	28.5 (24.0–33.5)	899	185	31.9 (29.7–34.2)	30.5 (25.7–35.7)
Delaware	1,247	189	25.3 (23.6–27.1)	22.3 (20.7–24.0)	381	62	33.6 (30.0–37.5)	34.6 (28.2–41.6)	443	67	38.5 (34.7–42.5)	37.5 (30.6–45.0)
District of Columbia	900	80	14.3 (13.2–15.5)	15.7 (14.7–16.9)	298	29	37.1 (33.1–41.3)	40.4 (32.1–49.4)	262	24	30.8 (27.1–34.8)	29.9 (23.1–37.7)
Florida	7,271	4,112	24.8 (23.6–26.0)	20.5 (19.5–21.5)	2,540	1,496	37.3 (34.7–40.0)	42.0 (36.9–47.3)	2,670	1,506	39.4 (36.7–42.1)	34.4 (29.8–39.3)
Georgia	1,826	1,734	22.3 (21.1–23.5)	21.0 (19.9–22.1)	606	562	32.9 (30.2–35.9)	29.2 (25.0–33.9)	743	697	43.7 (40.6–46.9)	39.9 (34.8–45.2)
Hawaii	1,942	232	21.0 (19.8–22.3)	19.0 (17.9–20.1)	411	51	22.2 (19.5–25.2)	27.3 (22.5–32.7)	463	66	30.1 (26.8–33.6)	33.4 (27.8–39.6)
Idaho	1,540	304	24.2 (22.7–25.7)	22.2 (20.8–23.6)	338	70	23.1 (20.4–26.1)	26.1 (21.2–31.7)	455	92	32.6 (29.3–36.0)	30.9 (25.4–37.1)
Illinois	1,726	2,405	24.5 (23.1–25.9)	22.4 (21.2–23.7)	388	633	26.4 (23.5–29.5)	28.4 (22.5–35.1)	495	688	30.5 (27.7–33.4)	24.9 (20.5–29.9)
Indiana	5,118	1,428	28.4 (27.5–29.3)	26.1 (25.3–27.0)	1,383	417	29.6 (28.0–31.3)	30.3 (27.5–33.3)	1,853	517	39.5 (37.7–41.4)	36.9 (33.8–40.2)
Iowa	2,309	588	24.6 (23.6–25.7)	22.0 (21.0–22.9)	434	123	21.1 (19.1–23.3)	22.4 (18.7–26.6)	698	179	32.7 (30.5–35.1)	28.4 (24.6–32.4)
Kansas	6,540	519	24.1 (23.4–24.7)	22.2 (21.6–22.8)	1,506	129	25.3 (23.9–26.7)	26.6 (24.3–29.0)	2,205	177	37.0 (35.4–38.5)	34.6 (32.0–37.4)
Kentucky	3,350	1,095	32.3 (30.7–33.8)	29.4 (28.0–30.9)	1,222	413	38.3 (35.5–41.2)	39.2 (35.0–43.5)	1,433	474	46.4 (43.5–49.3)	44.4 (40.0–48.8)
Louisiana	1,588	962	27.2 (25.7–28.8)	25.5 (24.1–26.9)	566	363	38.3 (35.2–41.5)	39.0 (34.3–44.0)	596	369	42.8 (39.5–46.1)	41.7 (36.8–46.9)
Maine	3,619	333	31.1 (29.8–32.5)	26.5 (25.2–27.9)	730	71	21.6 (19.6–23.7)	22.2 (18.6–26.2)	1,046	96	30.8 (28.6–33.1)	26.4 (22.8–30.4)
Maryland	4,907	1,146	24.9 (23.9–26.0)	22.8 (21.9–23.8)	1,140	304	26.8 (24.7–29.0)	31.8 (27.5–36.4)	1,520	367	35.2 (33.0–37.5)	33.5 (29.0–38.3)
Massachusetts	2,126	1,262	23.7 (22.2–25.4)	21.3 (20.0–22.8)	498	327	26.6 (23.3–30.1)	25.9 (20.9–31.7)	611	377	32.1 (28.5–35.9)	27.9 (22.3–34.2)
Michigan	3,953	2,338	30.5 (29.4–31.5)	27.1 (26.2–28.1)	1,079	749	32.4 (30.4–34.4)	34.8 (31.4–38.4)	1,217	781	35.2 (33.2–37.2)	36.2 (32.7–39.8)
Minnesota	4,269	833	19.8 (19.1–20.5)	17.8 (17.2–18.5)	811	165	20.2 (18.6–21.9)	22.1 (19.3–25.2)	1,319	253	32.7 (30.9–34.6)	31.2 (28.0–34.6)
Mississippi	1,915	657	29.3 (27.6–31.0)	27.2 (25.6–28.8)	709	272	42.0 (38.8–45.3)	45.2 (39.7–50.7)	725	256	43.1 (39.8–46.4)	41.6 (35.9–47.6)
Missouri	2,752	1,296	27.8 (26.5–29.1)	24.9 (23.8–26.1)	782	373	29.4 (27.0–31.9)	30.8 (26.6–35.4)	1,051	472	37.7 (35.1–40.3)	35.4 (30.9–40.1)
Montana	1,930	207	25.5 (24.0–26.9)	22.6 (21.3–24.0)	450	51	24.8 (22.1–27.8)	27.2 (22.8–32.1)	640	68	34.0 (31.0–37.2)	32.2 (27.5–37.2)
Nebraska	4,789	345	24.0 (23.1–25.0)	22.0 (21.1–22.9)	916	69	20.2 (18.4–22.1)	22.9 (19.4–26.8)	1,553	104	32.1 (30.1–34.2)	29.5 (25.9–33.2)
Nevada	1,112	462	20.3 (18.6–22.1)	18.5 (17.0–20.1)	286	136	29.8 (25.7–34.4)	31.0 (24.4–38.5)	322	150	33.9 (29.3–38.8)	30.1 (23.2–38.1)
New Hampshire	2,064	281	26.5 (25.1–28.1)	23.0 (21.6–24.4)	447	62	22.3 (19.9–24.9)	24.7 (19.6–30.7)	584	80	31.0 (28.2–34.1)	33.7 (27.2–40.9)
New Jersey	3,751	1,576	22.9 (21.8–24.1)	20.4 (19.4–21.4)	1,089	485	31.2 (28.6–33.9)	34.0 (29.0–39.4)	1,308	585	39.9 (37.1–42.7)	36.1 (31.0–41.5)
New Mexico	2,099	398	25.3 (24.0–26.8)	23.0 (21.7–24.4)	631	136	34.3 (31.3–37.5)	38.7 (33.4–44.2)	616	111	30.2 (27.4–33.1)	25.6 (21.9–29.8)
New York	3,509	3,445	22.6 (21.6–23.6)	20.4 (19.6–21.2)	976	1,083	32.0 (29.7–34.4)	32.8 (28.9–37.1)	1,086	1,085	34.8 (32.5–37.3)	33.3 (29.2–37.7)
North Carolina	1,477	1,921	24.4 (23.0–26.0)	22.1 (20.8–23.5)	517	695	36.9 (33.6–40.4)	43.6 (38.2–49.2)	524	663	36.0 (32.7–39.5)	36.4 (30.8–42.3)
North Dakota	2,307	141	24.3 (23.1–25.6)	23.3 (22.1–24.5)	378	27	19.3 (17.0–21.7)	21.7 (17.7–26.2)	749	46	35.0 (32.3–37.9)	35.9 (31.1–41.0)
Ohio	4,741	2,598	29.1 (28.0–30.2)	25.9 (24.9–27.0)	1,291	760	29.6 (27.5–31.7)	32.4 (28.7–36.4)	1,804	936	38.1 (36.0–40.3)	34.0 (30.3–37.8)
Oklahoma	2,423	814	27.8 (26.5–29.1)	26.0 (24.8–27.2)	667	257	32.4 (30.0–35.0)	32.9 (28.8–37.2)	947	314	41.4 (38.8–44.0)	37.4 (33.3–41.8)
Oregon	1,650	847	26.6 (25.2–28.0)	23.9 (22.7–25.2)	342	193	23.3 (20.8–25.9)	23.7 (19.8–28.1)	443	236	30.1 (27.3–33.0)	27.0 (22.9–31.5)
Pennsylvania	2,128	2,915	29.2 (27.8–30.6)	25.4 (24.2–26.7)	556	789	27.4 (24.9–30.0)	28.9 (24.9–33.4)	658	956	34.9 (32.1–37.8)	35.2 (30.3–40.4)
Rhode Island	1,968	229	27.4 (25.9–29.0)	24.7 (23.2–26.1)	508	64	28.1 (25.3–31.1)	33.2 (27.4–39.6)	616	74	35.1 (32.1–38.1)	34.0 (28.2–40.4)
South Carolina	4,286	1,082	28.0 (26.9–29.1)	24.9 (23.9–25.8)	1,340	371	34.9 (32.8–37.0)	38.5 (34.4–42.7)	1,445	362	36.1 (34.0–38.2)	35.5 (31.4–39.7)
South Dakota	2,077	145	22.2 (20.6–23.9)	20.0 (18.5–21.6)	468	32	22.6 (19.5–26.1)	22.4 (17.6–28.1)	621	46	33.0 (29.2–37.0)	28.6 (22.7–35.3)
Tennessee	2,107	1,540	30.1 (28.6–31.7)	27.4 (26.0–28.8)	710	547	36.1 (33.2–39.0)	36.7 (32.1–41.5)	799	562	40.5 (37.5–43.5)	37.0 (32.7–41.5)
Texas	3,818	4,438	21.3 (19.9–22.9)	20.8 (19.5–22.3)	1,127	1,572	36.0 (32.1–40.1)	35.5 (29.1–42.4)	1,474	1,697	41.8 (37.6–46.0)	38.7 (32.2–45.6)
Utah	2,512	414	19.3 (18.4–20.2)	20.2 (19.4–21.1)	531	89	22.0 (19.9–24.2)	22.4 (19.4–25.7)	695	110	27.6 (25.4–30.0)	25.9 (22.6–29.5)
Vermont	2,184	138	27.7 (26.4–29.1)	23.7 (22.6–24.9)	430	30	22.2 (19.9–24.6)	22.5 (18.6–26.9)	538	36	28.3 (25.8–31.0)	27.0 (22.5–31.9)
Virginia	3,184	1,628	25.1 (24.0–26.2)	23.1 (22.2–24.1)	835	481	29.9 (27.7–32.3)	30.7 (26.9–34.9)	1,064	567	36.6 (34.3–39.1)	36.6 (32.4–41.0)
Washington	4,154	1,359	24.1 (23.2–25.0)	22.3 (21.5–23.2)	808	294	21.9 (20.2–23.8)	22.1 (19.3–25.1)	1,002	327	25.5 (23.8–27.4)	23.7 (20.9–26.8)
West Virginia	2,501	561	39.2 (37.7–40.8)	34.6 (33.1–36.0)	856	206	37.3 (35.0–39.6)	37.5 (33.7–41.4)	996	226	41.4 (39.1–43.8)	39.0 (35.1–43.0)
Wisconsin	1,856	1,136	25.6 (24.2–27.1)	22.9 (21.6–24.2)	463	294	26.1 (23.4–29.0)	26.9 (22.5–31.8)	493	293	27.9 (24.9–31.0)	26.2 (21.2–31.9)
Wyoming	1,470	113	25.4 (23.9–26.9)	23.4 (22.1–24.8)	290	26	23.2 (20.4–26.1)	23.6 (19.1–28.9)	499	40	36.4 (33.2–39.6)	35.4 (30.2–41.1)
**State median**	**N/A**	**N/A**	**24.9**	**22.8**	**N/A**	**N/A**	**28.9**	**30.3**	**N/A**	**N/A**	**34.9**	**33.7**

**Abbreviations:** CI = confidence interval; N/A = not applicable.

* Estimates were age-standardized to the 2000 projected U.S. population aged ≥18 years using three groups (18–44, 45–64, and ≥65 years).

^†^ Respondents were classified as having arthritis if they responded “yes” to the question “Have you ever been told by a doctor or other health care professional that you have arthritis, rheumatoid arthritis, gout, lupus, or fibromyalgia?”

^§^ Severe joint pain was defined as a response of 7–10 to “Please think about the past 30 days, keeping in mind all of your joint pain or aching and whether or not you have taken medication. On a scale of 0 to 10 where 0 is no pain or aching and 10 is pain or aching as bad as it can be, during the past 30 days, how bad was your joint pain on average?”

^¶^ Physical inactivity was defined as reporting “no” to the question “During the past month, other than your regular job, did you participate in any physical activities or exercises such as running, calisthenics, golf, gardening, or walking for exercise?”

** Among adults aged ≥18 years with arthritis.

**FIGURE:**
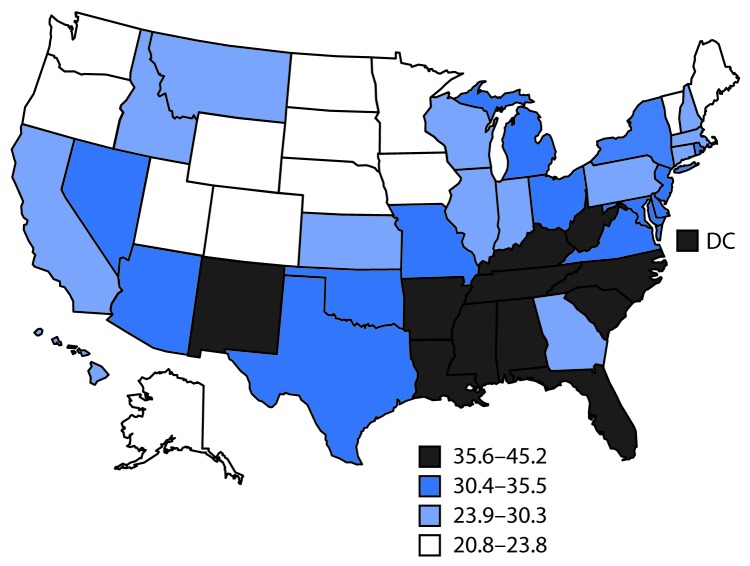
Age-standardized,* state-specific percentage of severe joint pain^†^ among U.S. adults aged ≥18 years with arthritis^§^ — Behavioral Risk Factor Surveillance System, United States, 2017 * Estimates were age-standardized to the 2000 projected U.S. population aged ≥18 years using three age groups (18–44, 45–64, and ≥65 years). ^†^ Severe joint pain was defined as a response of 7–10 to “Please think about the past 30 days, keeping in mind all of your joint pain or aching and whether or not you have taken medication. On a scale of 0 to 10 where 0 is no pain or aching and 10 is pain or aching as bad as it can be, during the past 30 days, how bad was your joint pain on average?” ^§^ Respondents were classified as having arthritis if they responded “yes” to the question “Have you ever been told by a doctor or other health care professional that you have arthritis, rheumatoid arthritis, gout, lupus, or fibromyalgia?”

## Discussion

The 2017 age-standardized prevalence of arthritis was highest in Appalachia and the Lower Mississippi Valley; prevalences of severe joint pain and physical inactivity among adults with arthritis were highest in southeastern states. Estimates for all three outcomes in 2017 were similar to those in 2015 (*5*). Except for age, urban-rural status, and sexual orientation, sociodemographic patterns for prevalences of severe joint pain and physical inactivity were similar and offer potential targets for interventions designed to reduce arthritis pain.

Joint pain is often managed with medications, which are associated with various adverse effects. The 2016 National Pain Strategy advises that pain-management strategies be multifaceted and individualized and include nonpharmacologic strategies,^††^ and the American College of Rheumatology recommends regular physical activity as a nonpharmacologic pain reliever for arthritis.^§§^ Although persons with arthritis report that pain, or fear of causing or worsening it, is a substantial barrier to exercising (*6*), physical activity is an inexpensive intervention that can reduce pain, prevent or delay disability and limitations, and improve mental health, physical functioning, and quality of life with few adverse effects (*7*,*8*).^¶¶^ Physical Activity Guidelines for Americans recommends that adults, including those with arthritis, engage in the equivalent of at least 150 minutes of moderate-intensity aerobic physical activity per week for substantial health benefits.*** Adults who are unable to meet the aerobic guideline because of their condition (e.g., those with severe joint pain) should engage in regular physical activity according to their abilities and avoid physical inactivity. Even small amounts of physical activity can improve physical functioning in adults with joint conditions (*9*). Most adults with arthritis pain can safely begin walking, swimming, or cycling to increase physical activity.

Arthritis-appropriate, evidence-based, self-management programs and low-impact, group aerobic, or multicomponent physical activity programs are designed to safely increase physical activity in persons with arthritis.^†††^^,^^§§§^ These programs are available nationwide and are especially important for those populations that might have limited access to health care, medications, and surgical interventions (e.g., those in rural areas, those with lower income, and racial/ethnic minorities). Physical activity programs including low-impact aquatic exercises (e.g., Arthritis Foundation Aquatic Program) and strength training (e.g., Fit and Strong!) can help increase strength and endurance. Participating in self-management education programs, such as the Chronic Disease Self-Management Program, although not physical activity–focused, is also beneficial for arthritis management and results in increased physical activity. Benefits of the Chronic Disease Self-Management Program include increased frequency of aerobic and stretching/strengthening exercise, improved self-efficacy for arthritis pain management, and improved mood (*10*). Adults with arthritis can also engage in routine physical activity through group aerobic exercise classes (e.g., Walk with Ease, EnhanceFitness, Arthritis Foundation Exercise Program, and Active Living Every Day).

The findings in this report are subject to at least three limitations. First, BRFSS data are self-reported and susceptible to recall, social desirability, and related biases. Second, low response rates for individual states might bias findings. Finally, institutional populations are excluded from sampling, meaning prevalences of studied outcomes are likely underestimated. Strengths include a measurement of joint pain and large sample size that allows analysis of detailed characteristics and subgroups.

Effective, inexpensive physical activity and self-management education programs are available nationwide and can help adults with arthritis be safely and confidently physically active. This report provides the most current state-specific and demographic data for arthritis, severe joint pain, and physical inactivity. These data can extend collaborations among CDC, state health departments, and community organizations to increase access to and use of arthritis-appropriate, evidence-based interventions to help participants reduce joint pain and improve physical function and quality of life.^¶¶¶^

**Summary**

**What is already known about this topic?**

Approximately one in four U.S. adults has arthritis. Severe joint pain and physical inactivity are common among adults with arthritis and are linked to poor mental and physical health outcomes.

**What is added by this report?**

In 2017, marked state-specific variations in prevalences of arthritis, severe joint pain, and physical inactivity were observed. Physical inactivity was more prevalent among persons with severe joint pain than among those with less pain.

**What are the implications for public health practice?**

State-specific data support efforts to promote participation in arthritis-appropriate, evidence-based self-management education and physical activity programs, which can reduce pain, increase physical activity and function, and improve mood and quality of life.

## Footnotes

* *Appalachia region:* all of West Virginia; parts of Alabama, Georgia, Kentucky, Maryland, New York, North Carolina, Ohio, Pennsylvania, South Carolina, and Virginia (https://www.arc.gov/appalachian_region/MapofAppalachia.asp). *Lower Mississippi Valley region:* Arkansas, Kentucky, Louisiana, Mississippi, Missouri, and Tennessee (https://www.mvd.usace.army.mil/Media/Publications/Our-Mississippi/About/Lower-Mississippi/).

†
https://www.cdc.gov/brfss/about/index.htm .

§ https://www.cdc.gov/brfss/annual_data/2017/pdf/2017-response-rates-table-508.pdf.

¶ http://citeseerx.ist.psu.edu/viewdoc/download?doi=10.1.1.684.5837&rep=rep1&type=pdf.

** Estimates were age-standardized to the 2000 projected U.S. population aged ≥18 years using three age groups: 18–44, 45–64, and ≥65 years. https://www.cdc.gov/nchs/data/statnt/statnt20.pdf.

†† https://iprcc.nih.gov/sites/default/files/HHSNational_Pain_Strategy_508C.pdf.

§§ http://mqic.org/pdf/2012_ACR_OA_Guidelines_FINAL.PDF.

¶¶ https://health.gov/paguidelines/second-edition/report/pdf/pag_advisory_committee_report.pdf.

*** https://health.gov/paguidelines/second-edition/pdf/Physical_Activity_Guidelines_2nd_edition.pdf.

††† https://www.cdc.gov/arthritis/interventions/physical-activity.html.

§§§ https://www.cdc.gov/arthritis/interventions/self_manage.htm.

¶¶¶ https://www.cdc.gov/arthritis/partners/index.htm.

## References

[1] Barbour KE, Helmick CG, Boring M, Brady TJ. Vital signs: prevalence of doctor-diagnosed arthritis and arthritis-attributable activity limitation—United States, 2013–2015. MMWR Morb Mortal Wkly Rep 2017;66:246–53. 10.15585/mmwr.mm6609e1 28278145 PMC5687192

[2] Barbour KE, Boring M, Helmick CG, Murphy LB, Qin J. Prevalence of severe joint pain among adults with doctor-diagnosed arthritis—United States, 2002–2014. MMWR Morb Mortal Wkly Rep 2016;65:1052–6. 10.15585/mmwr.mm6539a2 27711038

[3] Murphy LB, Hootman JM, Boring MA, Leisure time physical activity among U.S. adults with arthritis, 2008–2015. Am J Prev Med 2017;53:345–54. 10.1016/j.amepre.2017.03.017 28601405

[4] Research Triangle Institute. SUDAAN language manual, release 11. Research Triangle Park, NC: Research Triangle Institute; 2012.

[5] Barbour KE, Moss S, Croft JB, Geographic variations in arthritis prevalence, health-related characteristics, and management—United States, 2015. MMWR Surveill Summ 2018;67(No. SS-4) 10.15585/mmwr.ss6704a1 29543787 PMC5857191

[6] Wilcox S, Der Ananian C, Abbott J, Perceived exercise barriers, enablers, and benefits among exercising and nonexercising adults with arthritis: results from a qualitative study. Arthritis Rheum 2006;55:616–27. 10.1002/art.22098 16874785

[7] Kelley GA, Kelley KS, Hootman JM, Jones DL. Effects of community-deliverable exercise on pain and physical function in adults with arthritis and other rheumatic diseases: a meta-analysis. Arthritis Care Res (Hoboken) 2011;63:79–93. 10.1002/acr.20347 20824798

[8] Knapen J, Vancampfort D, Moriën Y, Marchal Y. Exercise therapy improves both mental and physical health in patients with major depression. Disabil Rehabil 2015;37:1490–5. 10.3109/09638288.2014.972579 25342564

[9] Dunlop DD, Song J, Lee J, Physical activity minimum threshold predicting improved function in adults with lower-extremity symptoms. Arthritis Care Res (Hoboken) 2017;69:475–83. 10.1002/acr.23181 28029748 PMC5521176

[10] Brady TJ, Murphy L, O’Colmain BJ, A meta-analysis of health status, health behaviors, and health care utilization outcomes of the Chronic Disease Self-Management Program. Prev Chronic Dis 2013. https://www.cdc.gov/pcd/issues/2013/12_0112.htm 10.5888/pcd10.120112 PMC3547675 23327828

